# Effects of Magnesium Oxide Nanoparticles Incorporation on Shear Bond Strength and Antibacterial Activity of an Orthodontic Composite: An In Vitro Study

**DOI:** 10.3390/biomimetics7030133

**Published:** 2022-09-14

**Authors:** Abdolrasoul Rangrazi, Maryam Sadat Daneshmand, Kiarash Ghazvini, Hooman Shafaee

**Affiliations:** 1 Dental Research Center, Mashhad University of Medical Sciences, Mashhad 91778-99191, Iran; 2 School of Dentistry, Mashhad University of Medical Sciences, Mashhad 91778-99191, Iran; 3 Antimicrobial Resistance Research Center, Mashhad University of Medical Sciences, Mashhad 91778-99191, Iran; 4 Department of Orthodontics, School of Dentistry, Mashhad University of Medical Sciences, Mashhad 91778-99191, Iran

**Keywords:** magnesium oxide, nanoparticles, shear bond strength, *Streptococcus mutans*, orthodontic composite

## Abstract

This study aimed to evaluate the effects of magnesium oxide (MgO) nanoparticle (NP) incorporation on shear bond strength (SBS) and antibacterial property of orthodontic composites. A total of 100 mounted premolar teeth were randomly divided into five groups. In group 1 (control), the brackets were bonded to the teeth using the GC Ortho Connect orthodontic composite, while the brackets of groups 2 to 5 were bonded by the GC Ortho Connect orthodontic composite that contained 0.5%, 1%, 2%, and 4% weight percentages (w/w) of MgO NPs, respectively and then the SBS was measured. In the following, we evaluated the antibacterial properties of the MgO NP-containing composite on *Streptococcus mutans* (*S. mutans*) bacteria by the direct contact test method. According to results, there were no significant changes in the SBS as the MgO NP concentration was increased up to 1%, while the SBSs of the 2% and 4% MgO NPs were decreased when compared to the other three groups. The outcomes of the direct contact test indicated the case of 1% as being the minimum ratio of MgO NPs, which almost caused the entire annihilation of the *S. mutans* bacteria. In conclusion, the orthodontic composite containing 1% MgO NPs can display a significant antibacterial effect against *S. mutans* bacteria without inducing any negative effect on the SBS.

## 1. Introduction 

Fixed orthodontic treatment is the most preferred mode of treatment for most types of malocclusions [1]. The complicated undercut of orthodontic devices makes it more difficult to maintain the cleansing of teeth, which can lead to the inducement of dental plaque accumulation, as well as cause alterations in the qualitative and quantitative profile of microbial flora and consequently increase the risk of white spot lesions (WSLs) and dental caries [2,3]. The data from the literature have pointed out the common occurrence of WSL in the course of fixed orthodontic treatments, with incidence and prevalence rates of 45.8% and 68.4%, respectively [4].

Standing as one of the main components involved in the fixed orthodontic treatment, brackets are used to transfer forces from the archwire to the dentition in order to facilitate teeth movement [5]. The existing WSLs and caries around and under brackets are known to be a major problem during fixed orthodontic treatments and can result in causing increased costs, delays in the treatment, and irreversible loss of tooth tissues [6,7]. To overcome this obstacle, patients are required to use products that contain fluoride, chlorhexidine, or casein phosphopeptides-amorphous calcium phosphate (CPP-ACP) [8,9,10,11,12]. However, researchers have attempted to focus on preventive strategies that would be independent of patient cooperation, which include coating the brackets with antimicrobial nanoparticles [13] or the addition of antimicrobial or remineralizing agents to the orthodontic composites that are exerted to bond the brackets to a tooth [14]. In recent years, researchers have tested the addition of different nanoparticles, such as Ag, CuO, TiO_2_, ZnO, chitosan, curcumin, cinnamon NPs, and ACP, into the resin of orthodontic composites [14,15,16,17,18,19,20,21]. Magnesium oxide (MgO) nanoparticles (NPs) attracted the interest of many in biomaterial science due to their economic precursor production and antimicrobial activity against various kinds of Gram-positive and Gram-negative bacteria, spores, and viruses [22]. In addition, MgO is also exerted for alleviating heartburn, stomach sore, bone regeneration, and tumor inhibition, as well as for the treatment of cancer including nanocryosurgery and hyperthermia [23]. As an important issue, it should be evaluated whether the applied modification to the orthodontic dental materials with antimicrobial agents would cause any significant negative effects to their mechanical properties [24,25,26]. Having an adequate bond strength between the enamel surface of the tooth and the bracket is an important factor for tolerating orthodontic forces and providing control over tooth movement. Failure in bracket bonding is a fatal obstacle in orthodontic treatments that can negatively affect the efficiency while increasing the duration and cost of the treatment [27,28].

Recent studies [22,29] have indicated the antibacterial and antibiofilm activities of MgO NPs against two cariogenic microorganisms, including *Streptococcus mutans* (*S. mutans*) and *Streptococcus sobrinus*. Due to its favorable properties, such as biocompatibility, good color, and antibacterial activity, MgO NPs stand as a suitable option for being added to orthodontic composites for the propose of improving their antibacterial features. To the best of our knowledge, there are no previous in vitro studies available on MgO NP-containing orthodontic composites. Thus, the main purpose of this work is to evaluate the effects of MgO NP addition on the SBS and the antibacterial activity against *S. mutans* as the most significant contributor to dental caries [30] of an orthodontic composite.

## 2. Materials and Methods

The first stage implicated the synthesis of MgO NPs, for which 5 g of magnesium chloride (Merck, Darmstadt, Germany) was dissolved in 50 mL of distilled water that contained a 10 mL solution of 1 N NaOH (Merck, Darmstadt, Germany). Then, the solution was stirred on a magnetic stirrer for 4 h to produce a magnesium hydroxide precipitate. The resulting suspension was centrifuged to obtain a magnesium hydroxide precipitate, which was washed with distilled water to go through another centrifugation process. Lastly, the precipitate was dried in a furnace to finish the production of magnesium oxide nanoparticles.

Our study included the usage of 100 extracted premolar teeth that were stored in 0.1% thymol solution for one week. The exerted teeth were free from caries, fillings, and endodontic treatment. The root of each tooth was mounted on acrylic blocks. Once all of the samples were cleaned with fluoride and oil free pumice, rinsed, and air dried, the buccal surfaces were acid-etched in accordance with the manufacturers’ instructions by using 37% phosphoric acid for 30 s. Afterwards, the teeth were washed for 15 s, dried, and then randomly divided into five groups (n = 20) as per the following:

Group 1 (control): Bonding with the orthodontic adhesive (GC Ortho Connect, GC Corp, Japan)

Group 2: Bonding with GC Ortho Connect containing 0.5% MgO NPs

Group 3: Bonding with GC Ortho Connect containing 1% MgO NPs

Group 4: Bonding with GC Ortho Connect containing 2% MgO NPs

Group 5: Bonding with GC Ortho Connect containing 4% MgO NPs

**Figure 1:**
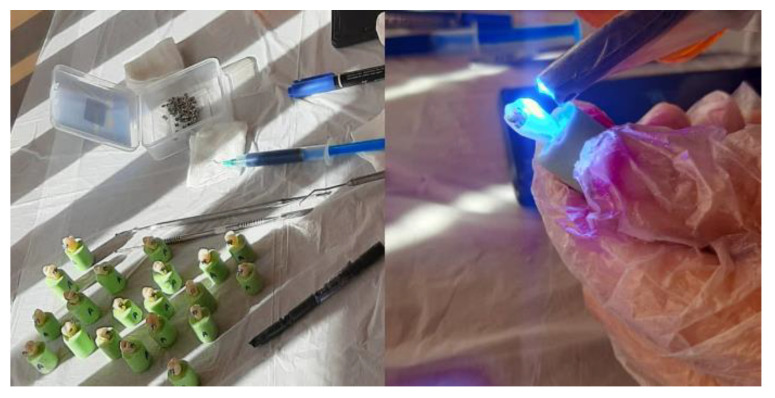
Bonding of the bracket to the enamel surface.

The bracket was placed on the buccal enamel surface to be light cured (Figure 1). All the specimens were stored in distilled water for 24 h at 37 °C in an incubator.

**Figure 2:**
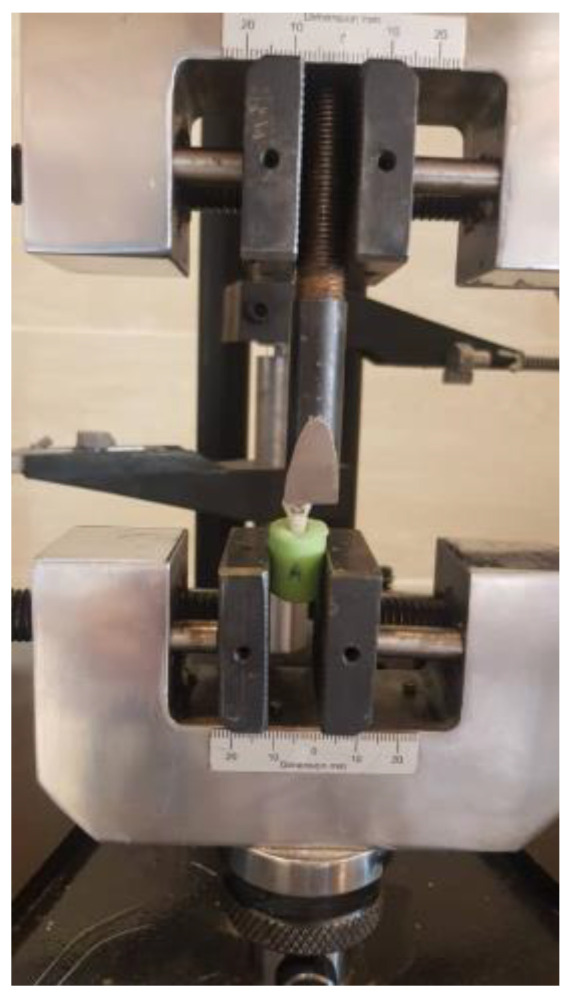
Positioning of a sample in the universal testing machine.

Then, they were thermocycled for 1000 cycles between the range of 5 °C and 55 °C, with a dwell time of 30 s. Each sample was fixed by the usage of a universal testing machine (STM20, SANTAM, Tehran, Iran) and the SBS was measured as the cross-head speed was adjusted to 1 mm/min (Figure 2). The shear load, recorded in newtons (N), was divided by the surface area of the bracket base (mm^2^) to calculate the value of SBS in megapascals (MPa).

**Figure 3:**
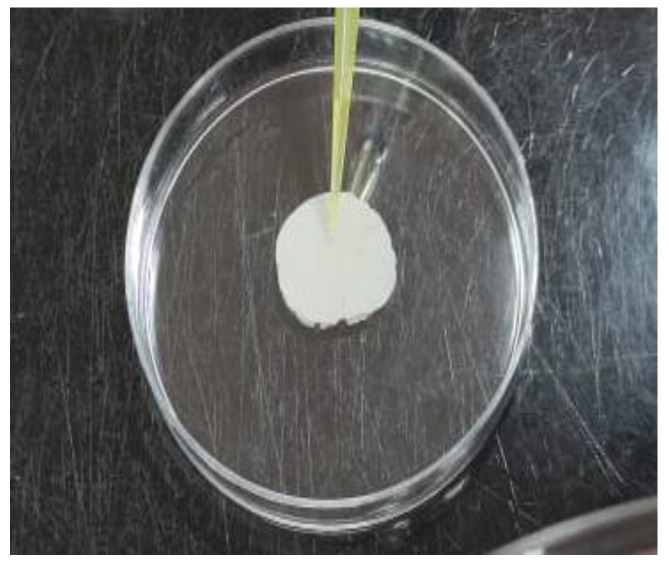
Direct contact test on a disk-shaped orthodontic composite sample.

A mold (15 mm in diameter and thickness of 2 mm) was used to prepare disk-shaped samples for each group (n = 3). Once the mold was filled with the composites that contained different percentages (1%, 2%, and 4%) of MgO NPs, a glass slide was placed on the filled hole to go through the light-curing process. The unmodified composite was considered as a control group. In this research, the following steps were performed to evaluate the antibacterial activity of MgO NPs by using the direct contact test method (Figure 3):

- The exerted bacteria in this study was *S. mutans* (PTCC No: 1683).
- The bacteria were procured from the Persian Type Culture Collection (PTCC) and cultured on Brain Heart Infusion Agar (BHIA) that was supplemented with 5% sheep blood plates to be incubated at 37 °C for 48 h.
- The bacteria cells were harvested and resuspended in sterile phosphate-buffered saline (PBS).
- The preparation of bacterial inoculum in the predetermined optical density of 0.5, known as the McFarland standard concentration, was done by dilution that implicated the usage of sterile phosphate-buffered saline (PBS).
- Then, the 0.5 McFarland standard was diluted in the ratio of 1:250 to achieve the bacterial cell concentration of 6 × 10^5^ CFU/mL.
- The samples were decontaminated by the application of ultraviolet (UV) radiation.
- An amount of 100 microliters of the prepared bacterial suspension was pipetted onto the surfaces of the sample. A laboratory glass slide was used as a negative control. Every inoculated surface was placed and kept in a sterile plate for 24 h.
- The samples’ surfaces were washed with liquid culture medium to recover their bacteria. Then, the washing results were cultured on the brain heart infusion agar (BHIA), which was supplemented with 5% sheep blood plates, to be incubated for 48 h.
- The number of colonies per plate was recorded to determine the number of viable bacteria.

**Figure 4:**
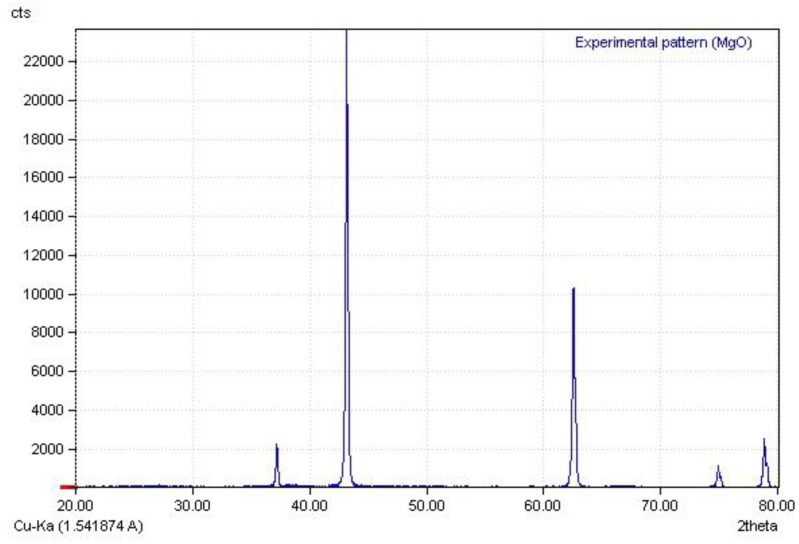
The X-ray diffraction diagram of MgO NPs.

The X-ray diffraction diagram of magnesium oxide nanoparticles is displayed in Figure 4, which is consistent of their X-ray diffraction pattern from other reference [31].

**Figure 5:**
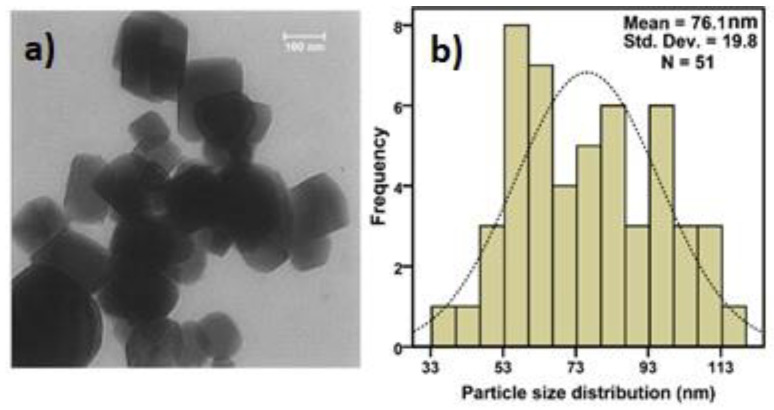
(**a**) TEM image and (**b**) size distribution of MgO NPs.

According to the exhibited TEM image and size distribution of MgO NPs in Figure 4, the average size of these particles is 76 nm (Figure 5).

## 3. Results

The descriptive statistics of SBS values for the five groups are shown in Table 1. The results of the ANOVA test displayed the existence of significant differences among the groups (*p* < 0.001), which was also indicated by the outcomes of Tukey’s test (*p* < 0.001).

As shown in Table 2, there were no significant differences between the control group, GC Ortho Connect containing 0.5% MgO NPs, and the GC Ortho Connect containing 1% MgO NPs, yet the SBS values of GC Ortho Connect composites containing 2% MgO NPs and 4% MgO NPs were significantly decreased. In other words, although increasing the concentration of MgO NPs up to 1% led to the inducement of insignificant changes in the SBS, a reduction was observed in the SBSs of 2% and 4% MgO NPs when compared to the other three groups.

Considering the results of the direct contact test (Table 3), the ratio of 1% is the minimum ratio of magnesium oxide NPs that almost caused the complete annihilation of *S. mutans* bacteria.

## 4. Discussions

In conformity to our results, although there were no significant decreases observed in the SBSs of composites up to the ratio of 1% of MgO NPs, the SBS values of 2% and 4% MgO NPs were notably reduced when compared to the control group. The important factors that can cause a reduction in the mechanical properties and SBS include the presence of MgO NPs as an impurity in the polymer matrix and the possibility of its agglomeration throughout the composite. These nanoparticles can interfere in the polymerization process and lower the degree of polymerization, which would subsequently result in weakening the mechanical properties. In addition, the agglomeration of nanoparticles in the composite can turn the agglomeration points into stress concentration points and reduce the SBS [32]. So far, there are no studies available on the effects of MgO NP addition on the SBS of orthodontic composites, while related investigations were performed on the impact of other nanoparticles, such as titanium dioxide, zinc oxide, chitosan, silver, and curcumin NPS, on these composites. Poosti et al. [17] investigated the effect of TiO_2_ NPs on the SBS of the Transbond XT orthodontic composite. According to their results, the addition of 1% of TiO_2_ NPs does not cause any significant reduction in the value of SBS. Moreover, Toodehzaeim et al. [16] reported the addition of CuO NPs to the Transbond XT orthodontic composite in the percentages of 0.01%, 0.5%, and 1%, which caused insignificant negative effects on the SBS of this composite. Similarly, the study results of Farzanegan et al. [32] indicated that the addition of 1% chitosan NPs and titanium dioxide led to an insignificant reduction in the SBS of the Transbond XT orthodontic composite. According to observations from Reddy et al. [33], having 1% silver oxide NPs, ZnO, and TiO_2_ caused a significant decrease in the SBS of the Transbond XT orthodontic composite. Furthermore, Felemban et al. [34] reported on the addition of ZrO_2_-TiO_2_ NPs to the Transbond XT orthodontic composite, which led to an increase in the SBS, compressive strength, and tensile strength of the orthodontic composite. In an in vitro study, Sodagar et al. [19] added curcumin NPs to the Transbond XT orthodontic composite in the proportions of 1%, 5%, and 10%. According to their observations, the case of 1% curcumin NPs did not induce any significant negative effects on the SBS of the Transbond XT orthodontic composite, which was in contrast to the results of higher ratios. The outcomes of Yaseen et al.’s research [21] indicated that the addition of cinnamon NPs to an orthodontic composite did not reduce the SBS.

The results of the direct contact test indicated 1% MgO NPs as being the minimum percentage of MgO NPs that is capable of almost destroying the entire *S. mutans* bacteria. In general, the high surface-to-volume ratio of nanoparticles leads to the inducement of active reactions with the bacterial membrane and consequently improves their antimicrobial properties [35]. MgO NPs were able to destroy the cell membrane and produce reactive oxygen species, resulting in the disruption of the activity of essential bacterial enzymes [36]. In addition, the high pH of these NPs plays an important role in regards to its antimicrobial activity [37]. Related studies have approved the antimicrobial activity of MgO NPs against Gram-negative and -positive bacteria, including *S. mutans* [22,38]. As it was mentioned, there are no records of MgO NP applications in orthodontic composites; however, the work of Noori et al. [22] reported the achievement of effective antimicrobial properties against *S. mutans* through the addition of MgO NPs to a restorative glass ionomer cement at a percentage of 1% or higher. Moreover, Toodehzaeim et al. [16] had similar observations, involving the addition of 1% CuO nanoparticles to Transbond XT orthodontic composite, which displayed antibacterial impacts against *S. mutans.* In conformity to the research results of Poosti et al. [17], the addition of 1% TiO_2_ NPs was able to significantly prevent the growth of *S. mutans*; similarly, Sodagar et al. [19] discovered that adding up to 10% of curcumin nanoparticles to Transbond XT orthodontic composite induced a reduction in the count of *S. mutans*. Furthermore, Pourhajibagher et al. [39] attempted to add 7.5% of curcumin–zinc oxide NPs to the Transbond XT orthodontic composite and succeeded in obtaining a significant antibacterial effect against *S. mutans*, S. *Sabrinus, and Lactobacillus acidophilus* bacteria. According to the observations by Yaseen et al. [21] on the same topic, the addition of 3% cinnamon NPs to the Transbond XT orthodontic composite was more effective than the addition of 1% cinnamon NPs to the orthodontic composites in terms of inducing antibacterial properties against *S. mutans*. Next to the mentioned in vitro studies, several clinical trial studies were conducted on the effects of nanoparticles on the antibacterial properties of orthodontic composites that are exerted for the bonding of brackets to teeth. In this regard, Jahanbin et al. [20] reported the observance of significant antimicrobial properties against *S. mutans* by the orthodontic composite that contained amorphous calcium phosphate nanoparticles during their study period (6 months). Furthermore, results of a clinical trial study by Rangrazi et al. [14] indicated that the addition of 1% TiO_2_ NPs and chitosan to an orthodontic composite caused a significant reduction in *S.mutans* during the time intervals of 2 months and 6 months in each of the lateral and premolar teeth.

## 5. Conclusions

It seems that MgO NPs induce antibacterial activity in orthodontic composites. Based on the current results, a 1% concentration is the minimum concentration of MgO NPs that has optimal antibacterial efficacy against *S. mutans* that does not cause any notable impacts on the SBS. It is necessary to conduct long-term clinical trials to evaluate the clinical performance of the addition of MgO NPs for improving the anticaries activity of the orthodontic composite.

## Figures and Tables

**Table 1:** Mean and standard deviation of the SBS in five different groups.

Groups	n	Mean (MPa)	Standard	F	*p*-Value
			Deviation		
**GC Ortho Connect (control)**	20	8.07	3.3	6.043	*p* < 0.001
**GC Ortho Connect +0.5% MgO**	20	7.17	2.01		
**GC Ortho Connect +1% MgO**	20	6.4	2.45		
**GC Ortho Connect +2% MgO**	20	5.55	1.94		
**GC Ortho Connect +4% MgO**	20	4.82	1.59		

**Table 2:** Mean and standard deviation of the SBS in five different groups.

(I) Group	(J) Group	*p*-Value
**GC Ortho Connect (control)**	**GC Ortho Connect +0.5% MgO**	0.738
	**GC Ortho Connect +1% MgO**	0.165
	**GC Ortho Connect +2% MgO**	0.008
	**GC Ortho Connect +4% MgO**	*p* < 0.001
**GC Ortho Connect +0.5% MgO**	**GC Ortho Connect +1% MgO**	0.834
	**GC Ortho Connect +2% MgO**	0.194
	**GC Ortho Connect +4% MgO**	0.017
**GC Ortho Connect +1% MgO**	**GC Ortho Connect +2% MgO**	0.784
	**GC Ortho Connect +4% MgO**	0.215
**GC Ortho Connect +2% MgO**	**GC Ortho Connect +4% MgO**	0.858

**Table 3:** The number of the colonies formed by *S. mutans* for each group.

Groups	CFU
**Glass Slab**	>100
**GC Ortho Connect (control)**	68
**GC Ortho Connect +0.5% MgO**	31
**GC Ortho Connect +1% MgO**	<10
**GC Ortho Connect +2% MgO**	<10
**GC Ortho Connect +4% MgO**	<10

## Data Availability

Data is contained within the article.
